# Data on *MECOM* rearrangement-driven chromosomal aberrations in myeloid malignancies

**DOI:** 10.1016/j.dib.2019.104025

**Published:** 2019-05-23

**Authors:** Zhenya Tang, Guilin Tang, Shimin Hu, Keyur P. Patel, C. Cameron Yin, Wei Wang, Pei Lin, Gokce A. Toruner, Chi Y. Ok, Jun Gu, Xinyan Lu, Joseph D. Khoury, L. Jeffrey Medeiros

**Affiliations:** a Department of Hematopathology, School of Health Professions, The University of Texas MD Anderson Cancer Center, Houston, TX 77030, USA; b Cytogenetic Technology Program, School of Health Professions, The University of Texas MD Anderson Cancer Center, Houston, TX 77030, USA; c Department of Pathology, Feinberg School of Medicine, Northwestern University, Chicago, IL 60611, USA

**Keywords:** *MECOM* rearrangement, Inversion, Insertion, Translocation, Fluorescence in situ hybridization (FISH)

## Abstract

Data in this article presents the results of conventional cytogenetics and fluorescence in situ hybridization (FISH) analyses in 129 patients with confirmed *MECOM* rearrangement (https://doi.org/10.1016/j.cancergen.2019.03.002) [1]. Generally, the *MECOM* rearrangement has arisen through translocation, inversion, and insertion and/or unknown mechanism. In addition to the typical chromosomal aberrations, inv(3)(q21q26.2) and t(3; 3)(q21; q26.6) [2–4], over 50% of cases presented here exhibit a wide spectrum of *MECOM* rearrangement-driven, atypical chromosomal aberrations, including inv(3) with breakpoint other than 3q21; t(1; 3); t(2; 3); t(3; 6); t(3; 8); t(3; 12); t(3; 17); t(3; 21) as well as an insertion of 3q26.2 into different chromosomes. These cases are thoroughly characterized by karyotyping, interphase-, metaphase-, map-back FISH and whole chromosomal painting (WCP) analyses.

**Table:** Specifications Table

Subject area	Biology
More specific subject area	tumor
Type of data	Table
How data was acquired	Microscope, The CytoVision 7.6 (Leica Biosystems)
Data format	Raw data
Experimental factors	Chromosomal analysis and FISH analysis on cultured bone marrow specimen by following standard clinical laboratory protocol
Experimental features	Conventional Cytogenetics and FISH analyses
Data source location	Houston, Texas, United State of America
Data accessibility	Data is with this article
Related research article	Z. Tang, G. Tang, S. Hu, K.P. Patel, C.C. Yin, W. Wang, P. lin, G.A. Toruner, C.Y. Ok, J Gu, X. Lu, J.D. Khoury and L.J. Medeiros. Deciphering the Complexities of MECOM Rearrangement-Driven Chromosomal Aberrations. Cancer Genet. 233–234 (2019) 21–31. https://doi.org/10.1016/j.cancergen.2019.03.002

**Table:** 

**Value of the data** • This dataset provides so far the largest number of cases from a single institute about *MECOM* rearrangement in myeloid malignancies [1]. Scientists and/or pathologists in the field of cytogenetic and genomic diagnostics can use these cases as references for complex cases involving chromosome 3 abnormalities. • The *MECOM* rearrangement has been confirmed by FISH analysis in all cases, comparing to cases with chromosome 3 abnormalities but without further confirmation of a *MECOM* rearrangement in previous studies [2], [3], [4], [5], [6]. • The majority of cases with atypical chromosomal abnormalities have been thoroughly analyzed by interphase-, metaphase-, map-back FISH and/or WCP. Therefore, the results, especially the ISCN description of all chromosomal aberrations, are accurate and reliable.

## 1 Data

This is an intensive characterization of complexities of chromosomal aberrations involving 3q26.2/*MECOM* rearrangement in 129 cases with a variety of myeloid malignancies. The majority of cases with atypical and/or complex karyotype has been thoroughly analyzed by interphase-, metaphase-, map-back FISH, and/or whole chromosome painting (WCP). In addition to the results of conventional cytogenetics and FISH analyses, other general clinical information including age, gender, pathologic diagnosis and outcomes, are also included (Table 1). We believe that all these cases can be practically useful for scientists and/or pathologists in the field of cytogenetic and genomic diagnostics. They can be used as references for all newly encountered case with a putative 3q26.2 abnormality/*MECOM* rearrangement.

**Table 1:** General information, karyotyping and MECOM FISH results in 129 cases of myeloid malignancies.

Case#	Sex	Age (y)	Diagnosis	D/A	FU (m)	Karyotype results	FISH results
1	M	70	AML	D	2	45,XY,inv(3)(p23q26.2),-7,add(8)(q24.1),del(20)(q11.2q13.1)[20]	ish inv(3)(p23q26.2)(3′MECOM+,5′MECOM+)[3].nucish(MECOMx2)(3′MECOM sep 5′MECOMx1)[153/200]
2	F	77	AML	D	1	43,X,-X,inv(3)(q13.3q26.2),del(5)(q22q35),add(6)(p21.1),-7,-17,-19,+mar[8]/43,idem,del(11)(p11.2)[3]/44,idem,+mar[3]/44,idem,i(5)(q10),+mar[2]/46,XX[4]	ish inv(3)(q13.3)(3′MECOM+)(q26.2)(5′MECOM+)[2].nucish(MECOMx2)(3′MECOM sep 5′MECOMx1)[172/200]
3	M	67	AML	D	10	45,XY,inv(3)(q21q26.2),-7[3].	ish inv(3)(q21)(5′MECOM+)(q26.2)(5′MECOM+,3′MECOM+)[3].nucish(3′MECOMx2,5′MECOMx3)(3′MECOM con 5′MECOMx2)[164/200]
4	M	72	AML	D	13	46,XY,inv(3)(q21q26.2)[20]	ish inv(3)(q21)(5′MECOM+)(q26.2)(3′MECOM+)[1].nucish(MECOMx2)(3′MECOM sep 5′MECOMx1)[76/200]
5	M	77	MDS, AML	D	4	46,XY,inv(3)(q21q26.2)[20]	ish inv(3)(q21)(5′MECOM+)(q26.2)(3′MECOM+)[1].nucish(MECOMx2)(3′MECOM sep 5′MECOMx1)[42/200]
6	M	74	MDS	D	32	46,XY,inv(3)(q21q26.2)[20]	nucish(MECOMx2)(3′MECOM sep 5′MECOMx1)[127/200]
7	M	55	AML	D	6	46,XY,inv(3)(q21q26.2)[19]/46,XY[1]	ish inv(3)(q21)(5′MECOM+)(q26.2)(3′MECOM+)[1].nucish(MECOMx2)(3′MECOM sep 5′MECOMx1)[158/200]nucish(MECOMx2)(3′MECOM sep 5′MECOMx1)[37/200]nucish(MECOMx2)(3′MECOM sep 5′MECOMx1)[39/200]
8	F	63	MDS	D	10	46,XX,inv(3)(q21q26.2)[20]	ish inv(3)(q21)(5′MECOM+)(q26.2)(3′MECOM+)[2].nucish(MECOMx2)(3′MECOM sep 5′MECOMx1)[114/200]
9	M	68	AML/MDS	D	2	45,X,-Y[15]/45,idem,inv(3)(q21q26.2)[5]	ish inv(3)(q21; q26)(3′MECOM+,5′MECOM+)[1].nucish(MECOMx2)(3′MECOM sep 5′MECOMx1)[24/200]
10	F	66	AML	D	1	46,XX,inv(3)(q21q26.2)[20]	nucish(MECOMx2)(3′MECOM sep 5′MECOMx1)[109/200]
11	M	41	AML	D	4	46,XY,inv(3)(q21q26.2)[15]/47,idem,+mar[2]/46,XY[3]	nucish(MECOMx2)(3′MECOM sep 5′MECOMx1)[36/200]
12	M	22	AML	D	4	45,XY,inv(3)(q21q26.2),-7[20]	nucish(MECOMx2)(3′MECOM sep 5′MECOMx1)[110/200]
13	F	29	t-MDS	A, CR (SCT)	37	46,XX,inv(3)(q21q26.2)[20]	nucish(MECOMx2)(3′MECOM sep 5′MECOMx1)[76/200]
14	M	74	AML/MDS	D	0	46,XY,inv(3)(q21q26.2)[20]	ish inv(3)(q21)(5′MECOM+)(q26.2)(3′MECOM+)[2].nucish(MECOMx2)(3′MECOM sep 5′MECOMx1)[100/200]
15	M	72	MDS	D	0	45,XY,inv(3)(q21q26.2),del(5)(q31q35),-7,del(12)(p11.2p13)[16]/45,idem,del(11) (p11.2)[2]/46,XY[2]	nucish(MECOMx2)(3′MECOM sep 5′MECOMx1)[102/200]
16	M	46	AML	D	5	46,XY,inv(3)(q21q26.2)	nucish(MECOMx2)(3′MECOM sep 5′MECOMx1)[146/200]
17	F	68	MDS, AML	D	3	44,XX,-3,inv(3)(q21q26.2),del(5)(q13q33),der(6)t(3; 6)(q23; p23),add(7)(q22),del(12)(p11.2p13),-20[17]/45,XX,inv(3)(q21q26.2),del(5)(q13q33),add(7)(q22),del(12)(p11.2p13),-20[3]	nucish(MECOMx2)(3′MECOM sep 5′MECOMx1)[138/200]
18	F	72	AML	D	3	46,XX,inv(3)(q21q26.2)[4]/46,idem,der(16)t(1; 16)(q21; q13)[15]/46,idem,del(X)(q21)[1]	nucish(MECOMx2)(3′MECOM sep 5′MECOMx1)[138/200]
19	M	32	AML	A, CR	21	46,XY,inv(3)(q21q26.2)[16]/46,XY[4]	nucish(MECOMx2)(3′MECOM sep 5′MECOMx1)[17/201
20	F	27	AML	D	4	43∼45,XX,add(2)(q32),add(3)(q27),inv(3)(q21q26.2),del(5)(q22q35),-7,del(14)(q22)[cp20]	nucish(MECOMx2)(3′MECOM sep 5′MECOMx1)[174/200]
21	F	49	AML	D	0	46,XX,inv(3)(q21q26.2)[2]/46,idem,der(X)del(X)(p22.1p22.3)add(X)(q24)[9]/45,idem,-7[9]	nucish(MECOMx2)(3′MECOM sep 5′MECOMx1)[32/200]
22	F	77	MDS	A, PR	3	46,XX,inv(3)(q21q26.2),r(7)[20]	nucish(MECOMx2)(3′MECOM sep 5′MECOMx1)[85/200]
23	M	77	AML	A, PR	17	47,XY,inv(3)(q21q26.2),del(7)(q22q34),+21[20]	nucish(3′MECOMx3,5′MECOMx2)(3′MECOM con 5′MECOMx2)[54/200]
24	M	91	MDS	D	1	47,XY,inv(3)(q21q26.2),+8[20]	nucish(MECOMx2)(3′MECOM sep 5′MECOMx1)[90/200]
25	F	57	AML	D	0	46,XX,inv(3)(q21q26.2)[15]/46,idem,add(2)(q11.2)[4]/46,idem,der(2)t(2; 11)(q11.2; q13)[1]	ish inv(3)(q21)(3′MECOM+)(q26.2)(5′MECOM+)[2].nucish(MECOMx2)(3′MECOM sep 5′MECOMx1)[145/200]
26	M	80	MDS	D	1	45,XY,inv(3)(q21q26.2),-7[20]	nucish(MECOMx2)(3′MECOM sep 5′MECOMx1)[131/200]
27	F	73	polycythemia vera with MF-3	D	0	46,XX,t(12; 18)(q13; q21.1)[4]/46,idem,add(13)(q12)[6]/46,idem,inv(3)(q21q26.2)[4]/46,idem,del(13)(q12q22)[3]/46,idem,add(6)(p21)[2]/45,idem,dic(6; 11)(p21; p11.2),add(7)(p22)[1]	nucish(MECOMx2)(3′MECOM sep 5′MECOMx1)[24/200]/(MECOMx4)[17/200]
28	F	39	AML	A, PR (SCT)	11	46,XX,inv(3)(q21q26.2)[20]	nucish(MECOMx2)(3′MECOM sep 5′MECOMx1)[140/200]
29	M	48	AML	A, CR (SCT)	7	45,XY,inv(3)(q21q26.2),-7[19]/46,XY[1]	nucish(MECOMx2)(3′MECOM sep 5′MECOMx1)[117/200]
30	M	17	AML	A, NR	7	45,XY,inv(3)(q21q26.2),-7[18]/45,idem,+2mar[cp2]	ish inv(3)(q21)(3′MECOM+)(q26.2)(5′MECOM+)[2].nucish(MECOMx2)(3′MECOM sep 5′MECOMx1)[146/200]
31	M	78	AML	D	4	45,XY,inv(3)(q21q26.2),-7,inv(9)(p12q13)[9]/46,XY,inv(9)(p12q13)[11]	nucish(MECOMx2)(3′MECOM sep 5′MECOMx1)[94/200]
32	F	70	MDS	D	1	46,XX,inv(3)(q21q26.2)[20]	nucish(MECOMx2)(3′MECOM sep 5′MECOMx1)[103/200]
33	M	52	AML	A, PR	4	46,XY,inv(3)(q21q26.2),t(7; 9)(p15; p23)[20]	nucish(MECOMx2)(3′MECOM sep 5′MECOMx1)[124/200]
34	M	67	AML	D	2	45,X,-Y,inv(3)(q21q26.2)[20]	nucish(MECOMx2)(3′MECOM sep 5′MECOMx1)[147/200]
35	M	65	t-MDS; metastatic PROSTATIC ADENOCARCINOMA	A, PR	2	42∼49,Y,add(X)(p22),inv(3)(q21q26.2),-4,-5,del(5)(q31q35),-7,-8,-9,add(11)(p15),-12,add(12)(p13),+14,+15,-16,del(17)(p12),-19,-21,+2∼8 mar[cp20]	nucish(MECOMx2)(3′MECOM sep 5′MECOMx1)[15/200]
36	F	45	AML	A, PR	1	46,XX,inv(3)(q21q26.2)[14]/46,XX[6]	nucish(MECOMx2)(3′MECOM sep 5′MECOMx1)[68/200]
37	F	53	AML	A, NR	0	45,XX,inv(3)(q21q26.2),-7[19]/46,idem,dup(1)(q25q32),-14,-16,+3mar[1]	nucish(MECOMx2)(3′MECOM sep 5′MECOMx1)[135/200]
38	M	63	CML, BP	A, PR	11	46,XY,t(9; 22)(q34; q11.2)[5]/46,idem,inv(3)(q21q26.2)[10]/46,idem,inv(16)(p13.1q22)[5]	nucish(MECOMx2)(3′MECOM sep 5′MECOMx1)[43/200]
39	F	45	CML, CP	D	5	46,XX,inv(3)(q21q26.2),t(9; 22)(q34; q11.2)[4]/46,idem,add(4)(q22),+12,-17,-17,+21[1]/46,XX[5]	nucish(MECOMx2)(3′MECOM sep 5′MECOMx1)[126/200]
40	M	61	CML, BP/AML	D	12	46,XY,inv(3)(q21q26.2),t(6; 13)(q13; q34),t(9; 22)(q34; q11.2),t(16; 17)(q13; q25)[19]/46,XY[1]	nucish(MECOMx2)(3′MECOM sep 5′MECOMx1)[104/200]
41	F	46	CML, BP	A, PR	1	45,XX,inv(3)(q21q26.2),-7,t(9; 22)(q34; q11.2),-15,+mar[10]/46,idem,+mar[7]/46,idem,+8[1]/45,XX,t(1; 2)(p34; p25),inv(3)(q21q26.2),-7,t(9; 22)[2]	nucish(MECOMx2)(3′MECOM sep 5′MECOMx1)[115/200]
42	M	37	CML, CP	A, PR	8	46,XY,inv(3)(q21q26.2),t(9; 22)(q34; q11.2)[17]/46,XY[1]	nucish(MECOMx2)(3′MECOM sep 5′MECOMx1)[171/200]
43	M	41	t-AML	D	9	46,XY,inv(3)(q21q26.2)[3]/45,idem,-7[16]/46,XY[1]	nucish(MECOMx2)(3′MECOM sep 5′MECOMx1)[53/200]nucish(MECOMx2)[200]nucish(MECOMx2)(3′MECOM sep 5′MECOMx1)[15/200]
44	F	56	AML/MDS	D	5	46,XX,inv(3)(q21q26.2)x2[1]/45,XX,idem,-7[14]/46,XX[5]	ish inv(3)(q21)(5′MECOM+)(q26.2)(3′MECOM+)x2[3].nucish(MECOMx2)(3′MECOM sep 5′MECOMx2)[85/200]
45	F	70	AML	D	4	46,XX,inv(3)(q21q26)x2[16]/46,idem,+1,der(1; 15)(q10; q10)[4]	ish inv(3)(q21)(3′MECOM+)(q26.2)(5′MECOM+)x2[2].nucish(MECOMx2)(3′MECOM sep 5′MECOMx2)[40/200]
46	M	44	MDS, AML	A, PR	3	43∼45,XY,inv(3)(p21q28),inv(3)(q21q26.2),del(5)(q22q35),-7,-12,der(12; 14)(q10; q10),-14,-17,del(17)(p12),+1∼3mar[cp20]	ish inv(3)(q21)(3′MECOM+)(q26.2)(5′MECOM+)[2]/inv(3)(p21q28)(MECOM+)[1].nuc ish(MECOMx2)(3′MECOM sep 5′MECOMx1)[44/200]
47	F	46	CML, BP	A, PR	5	46,XX,add(2)(q33),inv(3)(q26.2q28),t(9; 22)(q34; q11.2)[4]/47,idem,+8[8]	nucish(MECOMx2)(3′MECOM sep 5′MECOMx1)[51/200]
48	F	83	MDS	D	2	44,XX,der(3)del(3)(p25)inv(3)(q21q26.2)add(3)(q26.2),-5,-7,-12,add(17)(p11.2),i(22)(q10),+r[6]/43,XX,der(3)inv(3)(q21q26.2)add(3)(q26.2),-5,-7,-15,add(17)(p11.2),i(22)(q10),+mar[6]/42∼45,XX,der(3)inv(3)(q21q26.2)add(3)(q26.2),-5,-7,-12,add(17)(p11.2),-20,i(22)(q10),+mar[cp6]/46,XX[2]	ish der(3)inv(3)(q21)(3′MECOM-)(q26.2)(5′MECOM+)add(3)(q26.2)[5].nucish(3′MECOMx1,5′MECOMx2)(3′MECOM con 5′MECOMx1)[86/200]
49	M	65	MDS	D	11	45,XY,der(3)ins(3; ?)(q21; ?)inv(3)(q21q26.2),der(5)del(5)(q13q33)t(5; 11)(q35; q22),-7,der(11)t(5; 11),inv(12)(p12q21),add(13)(q34),-15,del(20)(q11.2q13.3),+mar,+2∼5dmin[17]/46,XY[2]	ish der(3)ins(3; ?)(q21; ?)inv(3)(q21)(5′MECOM+)q26.2)(3′MECOM+)[2].nuc ish(MECOMx2)(3′MECOM sep 5′MECOMx1)[21/200]
50	F	57	AML	D	11	46,XX,der(3)t(3; 11)(q13.2; q21),der(11)t(3; 11)inv(3)(q21q26.2),del(12)(p13)[18]/46,idem[cp2]	ish der(3)t(3; 11)(q13.2; q21)(MECOM-),der(11)t(3; 11)inv(3)(q21)(3′MECOM+)(q26.2)(5′MECOM+)[2].nucish(MECOMx2)(3′MECOM sep 5′MECOMx1)[145/200]. MLL FISH negative
51	M	69	AML	D	4	46,XY,der(3)t(3; 14)(p13; q32)inv(3)(q21q26.2),der(14)t(3; 14)[18]/46,idem,del(7)(p12)[2]	ish der(3)t(3; 14)(p13; q32)inv(3)(q21)(5′MECOM+)(q26.2)(3′MECOM+)[4].nuc ish(MECOMx2)(3′MECOM sep 5′MECOMx1)[132/200]
52	M	56	MDS	D	8	46,XY,-7,+mar[1]/46,sl,der(3)t(3; 3)(p21; q13),der(3)t(3; 3)inv(3)(q21q26.2)[9]/47,sdl,+mar[7]/47∼48, sl,+1∼2mar[cp3]	ish der(3)t(3;3)(p21; q13)(MECOM-),der(3)t(3;3)inv(3)(q21q26.2)(3′MECOM+,5′MECOM+,MECOM+)[2].nuc ish(MECOMx2)(3′MECOM sep 5′MECOMx1)[107/200]
53	F	45	AML/MDS	A, CR (SCT)	55	46,XX,t(1; 3)(q32; q26.2)[20]	ish t(1; 3)(q32; q26)(5′MECOM+; 3′MECOM+)[2].nucish(MECOMx2)(3′MECOM sep 5′MECOMx1)[180/200]nucish(MECOMX2)[200]
54	F	65	donor cell AML	A, PR	1	//46,XY,der(2)t(2; 3)(q21; q26.2),der(3; 3)(q10; q10)add(3)(q26.2)t(2; 3),del(20)(q11.2q13.3)[12]	ish der(2)t(2; 3)(p21; q26.2)(5′MECOM+),der(3; 3)(q10; q10)add(3)(q26.2)(3′MECOM+)t(2; 3)(3′MECOM+)[1].nuc ish(3′MECOMx3,5′MECOMx2)(3′MECOM con 5′MECOMx1)[176/200]/(MECOMx2)(3′MECOM sep 5′MECOMx1)[24/200]
55	F	77	MDS + CML	D	1	47,XX,t(2; 3)(p16; q26.2),del(7)(q22q34),t(9; 22)(q34; q11.2),+21[20]	ish t(2; 3)(p16; q26.2)(5′MECOM+; 3′MECOM+)[3].nucish(MECOMx2)(3′MECOM sep 5′MECOMx1)[191/200]
56	F	46	MDS/AML	D	23	46,XX,t(2; 3)(p21; q26.2)[20]	ish t(2; 3)(5′MECOM+; 3′MECOM+)[2].nucish(MECOMx2)(3′MECOM sep 5′MECOMx1)[185/200]
57	F	69	MDS/CMML	D	12	46,XX,t(2; 3)(p21; q26.2)[20]	ish t(2; 3)(p21; q26.2)(5′MECOM+; 3′MECOM+)[5].nucish(MECOMx2)(3′MECOM sep 5′MECOMx1)[175/200]
58	F	80	MDS	D	4	45,XX,t(2; 3)(p21; q26.2),-7[2]/46,t(2; 3),r(7)(p21q11.2)[2]/46,XX[16]	ish t(2; 3)(p21; q26.2)(5′MECOM+; MECOM+)[1].nucish(3′MECOMx2,5′MECOMx3)(3′MECOM con 5′MECOMx2)[35/200]
59	F	56	CML, BP	D	8	45,XX,t(2; 3)(p21; q26.2),-7,t(9; 22)(q34; q11.2)[19]/45,idem,add(6)(q21)[1]	ish t(2; 3)(p21; q26.2)(5′MECOM+; 3′MECOM+)[2].nucish(MECOMx2)(3′MECOM sep 5′MECOMx1)[190/200]
60	M	65	MDS/AML	D	13	46,XY,t(2; 3)(p23; q26.2)[20]	ish t(2; 3)(5′MECOM+; 3′MECOM+)[2].nucish(MECOMx2)(3′MECOM sep 5′MECOMx1)[194/200]
61	M	63	AML	D	1	46,XY,t(2; 3)(p23; q26.2),r(7)[20]	ish t(2; 3)(p23; q26.2)(5′MECOM+; MECOM+)[2].nucish(3′MECOMx2,5′MECOMx3)(3′MECOM con 5′MECOMx2)[172/200]
62	M	53	AML	D	11	46,XY,t(2; 3)(p23; q26.2)[19]/47,idem,+21[1	nucish(3′MECOMx2,5′MECOMx3)(3′MECOM con 5′MECOMx2)[158/200]
63	F	38	MDS	D	6	46,XX,t(2; 3)(p23; q26.2)[5]/46,idem,-5,+r[13]/46,XX[2]	nucish(3′MECOMx2,5′MECOMx3)(3′MECOM con 5′MECOMx2)[172/200]nucish(MECOMx2)(3′MECOM sep 5′MECOMx1)[123/200]
64	F	57	AML	A, PR (SCT)	8	46,XX,t(2; 3)(p23; q26.2),del(5)(q22q35)[20]	ish t(2; 3)(p23; q26.2)(5′MECOM+; MECOM+)[2].nucish(3′MECOMx2,5′MECOMx3)(3′MECOM con 5′MECOMx2)[197/200]
65	M	39	CML, CP	A, PR	5	47,XY,+8[4]/46,X,-Y,+8[12]/46,XY,t(2; 3)(p23; q26.2),t(9; 22)(q34; q11.2)[4]	ish t(2; 3)(p23; q26.2)(5′MECOM+; MECOM+)[1].nucish(3′MECOMx2,5′MECOMx3)(3′MECOM con 5′MECOMx2)[11/200]
66	F	51	MDS	D	22	46,XX,t(2; 3)(p23; q26.2)[17]/47,idem,+mar[2]/46,idem,der(18)t(1; 18)(q21; p11.2)[1]	nucish(3′MECOMx2,5′MECOMx3)(3′MECOM con 5′MECOMx2)[70/200]ish(3′MECOMx2,5′MECOMx3)(3′MECOM con 5′MECOMx2)[38/200]nucish(MECOMx2)(3′MECOM sep 5′MECOMx1)[95/200]nucish(MECOMx2)(3′MECOM sep 5′MECOMx1)[180/200]nucish(MECOMx2)(3′MECOM sep 5′MECOMx1)[178/200]
67	M	52	AML	A, NR	5	45,XY,der(3)t(3; 3)(q21; q26.2),der(3)t(5; 17)(q22; p12)t(3; 5)(p12; q13)t(3; 3),t(4; 18)(p16; q21.1),-5,der(7)del(7)(p12)del(7)(q11.2),der(17)t(3; 7)(p22; q22)t(7; 17)(q32; p11.2),-20,+mar[20]	ish der(3)t(3; 3)(q21; q26.2)(5′MECOM+), der(3)t(5; 17)(q22; p12)t(3; 5)(p12; q13)t(3; 3)(MECOM+,MECOM+)[2].nuc ish(3′MECOMx2,5′MECOMx3)(3′MECOM con 5′MECOMx2)[200]
68	M	73	AML	D	3	46,XY,t(3; 3)(q21; q26.2)[11]/45,idem,del(6)(q21),-7[7]/46,idem,del(7)(q22)[1]/45,idem,del(16)(p11.1)[1]	ish t(3; 3)(q21; q26.2)(3′MECOM+; MECOM+,5′MECOM+)[4].nucish(MECOMx2)(3′MECOM sep 5′MECOMx1)[190/200]
69	F	73	MDS	D	5	46,XX,del(5)(q13q33)[2]/46,idem,t(3; 3)(q21; q26.2),del(7)(q22q35)[18]	ish t(3; 3)(q21; q26.2)(5′MECOM+; MECOM+,3′MECOM+)[2].nucish(MECOMx2)(3′MECOM sep 5′MECOMx1)[174/200]
70	F	74	AML	D	8	44∼45,XX,t(3; 3)(q21; q26.2),-7[cp3]/46,XX[17].	nucish(MECOMx2)(3′MECOM sep 5′MECOMx1)[36/200]
71	M	18	AML	A, NR	7	45,XY,t(3; 3)(q21; q26.2),-7[18]/46,XY[2]	nucish(MECOMx2)(3′MECOM sep 5′MECOMx1)[36/200]
72	M	65	AML	A, NR	4	43,XY,der(1)inv(1)(p22q32)add(1)(q32),t(3; 3)(q21; q26.2),del(5)(q31q35),-7,del(12)(p11.2),-14,-16,add(19)(q13.3),add(20)(q11.2)[20]	nucish(MECOMx2)(3′MECOM sep 5′MECOMx1)[74/200]
73	M	40	AML	A, NR	0	45,XY,t(3; 3)(q21; q26.2),-7[8]/45,idem,+1,der(1; 15)(q10; q10),del(14)(q11.2q24)[2]/46,XY[1]	nucish(MECOMx2)(3′MECOM sep 5′MECOMx1)[62/200]
74	M	58	AML	D	20	46,XY,t(3; 3)(q21; q26.2)[3]//46,XX[5]	ish t(3; 3)(3′MECOM+; MECOM+)[1]/nucish(3′MECOMx2,5′MECOMx3)(3′MECOM con 5′MECOMx2)[40/200]
75	F	40	AML	D	0	47,XX,t(3; 3)(q26.2; q21),t(9; 22)(q34; q11.2),del(11)(p11.2p12),+19[11]/47,idem,del(8)(q12q23)[1]/47,XX,t(3; 3),del(8)(q12q23),t(9; 22)(q34; q11.2),del(11)(p12p15),der(19)t(19; 22)(p13.1; q11.2)t(9; 22),+19[4]/47∼48,XX,t(3; 3),der(7)t(7; 22)(q11.2; p11.2),der(9)t(9; 22),+19,der(22)t(7; 22)t(9; 22),+der(22)t(9; 22),+1∼2mar[cp5]	nucish(MECOMx2)(3′MECOM sep 5′MECOMx1)[193/200]
76	M	19	AML	D	12	46,XY,t(3; 6)(q26.2; p23),der(4)t(3; 4)(q12; p16),del(5)(q13q33),add(18)(q23)[3]/46,XY,t(3; 6)(q26.2; p23),der(4)t(3; 4)(q12; p16),add(5)(q21)[1]/46,XY,t(3; 6)(q26.2; p23),der(4)t(3; 4)(q12; p16),add(5)(q21),der(7)t(3; 7)(q26.2; q36)[1]/46,XY[20]	ish t(3; 6)(q26.2; p23)(3′MECOM+; 5′MECOM+),der(4)t(3; 4)(q12; p16)(MECOM+),der(7)t(3; 7)(q26.2; q36)(MECOM+)[1].nuc ish(3′MECOMx3,5′MECOMx4)(3′MECOM con 5′MECOMx3)[11/200]
77	F	64	AML	A, NR	0	46,XX,t(3; 6)(q26.2; q25)[20]	ish t(3; 6)(q26.2; q25)(3′MECOM+; 5′MECOM+)[2].nucish (MECOMx2)(3′MECOM sep 5′MECOMx1)[193/200]
78	M	67	MDS	D	22	46,XY,t(3; 6)(q26.2; q26)[23]	ish t(3; 6)(q26.2; q26)(3′MECOM+; 5′MECOM+)[2].nucish(MECOMx2)(3′MECOM sep 5′MECOMx1)[195/200]
79	M	70	AML	D	2	46,XY,t(3; 6)(q26.2; q26)[20]	ish t(3; 6)(q26.2; q26)(3′MECOM+; 5′MECOM+)[2].nucish(MECOMx2)(3′MECOM sep 5′MECOMx1)[190/200]
80	M	66	AML	D	2	46,XY,t(3; 8)(q26.2; q24.1)[5]/45,idem,-7[10]/43∼45,idem,dup(1)(q12q25),-7,-13[cp5]	ish t(3; 8)(q26.2; q24.1)(3′MECOM+; 5′MECOM+)[2].nucish(MECOMx2)(3′MECOM sep 5′MECOMx1)[126/200]nucish(MECOMx2)(3′MECOM sep 5′MECOMx1)[16/200] (AB-14-24500)
81	F	75	MDS	A, NR	7	46,XX,t(3; 8)(q26.2; q24.1)[20]	nucish(MECOMx2)(3′MECOM sep 5′MECOMx1)[186/200]
82	M	21	MDS, Fanconi's anemia	D	8	46,XY,t(3; 8)(q26.2; q24.1),der(7)t(7; 8)(q22; q13)t(3; 8),der(22)t(1; 22)(q25; q13)[6]/46,idem,der(2)t(2; 21)(q37; q11.2)[11]/46,idem,del(2)(q31)[3]	ish t(3; 8)(q26.2; q24.1)(3′MECOM+; 5′MECOM+),der(7)t(7; 8)(q22; q13)t(3; 8)(5′MECOM+)[2].nuc ish(3′MECOMx2,5′MECOMx3)(3′MECOM con 5′MECOMx1)[184]
83	M	63	AML	D	0	46,XY,t(3; 8)(q26.2; q24.2)[16]/46,XY[4]	ish t(3; 8)(q26.2; q24.2)(3′MECOM+; 5′MECOM+)[2].nucish(MECOMx2)(3′MECOM sep 5′MECOMx1)[188/200]
84	M	59	AML	D	3	46,XY,t(3; 8)(q26.2; q24.2)[20]	ish t(3; 8)(q26.2; q24.2)(3′MECOM+; 5′MECOM+)[2].nucish(MECOMx2)(3′MECOM sep 5′MECOMx1)[166/200]
85	F	65	AML	D	1	45,XX,t(3; 8)(q26.2; q24),-7[17]/46,idem,+21[1]/47,idem,+21,+22[2]	ish t(3; 8)(3′MECOM+; 5′MECOM+)[3].nucish(MECOMx2)(3′MECOM sep 5′MECOMx1)[195/200]
86	M	48	t-AML	D	0	46,XY,t(3; 10; 21)(q26.2; p14; q22)[20]	ish t(3; 10; 21)(q26.2; p14; q22)(MECOM+; 5′MECOM+; MECOM-)[3].nucish(3′MECOMx2,5′MECOMx3)(3′MECOM con 5′MECOMx2)[166/200].isht(3; 10; 21)(q26.2; p14; q22)(wcp21+; wcp21-;wcp21+)[3]
87	M	61	AML	D	12	45,XY,t(3; 12)(q26.2; p13),-7[2]/46,XY,idem,+mar[10]/45,idem,-5,add(8)(p23),+mar[7]	ish t(3; 12)(q26.2; p13)(3′MECOM+; 5′MECOM+)[2].nucish(MECOMx2)(3′MECOM sep 5′MECOMx1)[192/200]
88	M	45	AML	A, CR (SCT)	70	46,XY,t(3; 12)(q26.2; p13)[10]/46,XY[10]	nucish(MECOMx2)(3′MECOM sep 5′MECOMx1)[54/200]
89	F	45	AML	A, PR (SCT)	36	46,XX,t(3; 12)(q26.2; p13)[19]/46,XX[1]	ish t(3; 12)(q26.2; p13)(3′MECOM+; 5′MECOM+)[2].nucish(MECOMx2)(3′MECOM sep 5′MECOMx1)[176/200]nucish(MECOMx2)[200] (AB-15-11961)
90	F	33	AML	D	5	45,XX,t(3; 12)(q26.2; p13),-7[20]	ish t(3; 12)(q26.1; p13)(3′MECOM+; 5′MECOM+)[1].nucish(MECOMx2)(3′MECOM sep 5′MECOMx1)[200]
91	F	63	MDS	A, PR (SCT )	16	46,XX,t(3; 12)(q26.2; p13)[2]/46,XX[23]	nucish(MECOMx2)(3′MECOM sep 5′MECOMx1)[131/200]
92	F	60	AML	D	2	46,XX,t(3; 12)(q26.2; p13)[3]/41∼45,idem,-22[cp6]	ish t(3; 12)(q26.2; p13)(5′ETV6+; 3′ETV6+)[2].nucish(ETV6x2)(5′ETV6 sep 3′ETV6x1)[120/200].nucish(MECOMx2)(3′MECOM sep 5′MECOMx1)[106/200]
93	F	38	AML	A, NR	4	46,XX,t(3; 12)(q26.2; p13),del(7)(q22q36)[18]/46,idem[cp2]	ish t(3; 12)(q26.2; p13)(MECOM+; 5′MECOM+)[2].nucish(3′MECOMx2,5′MECOMx3)(3′MECOM con 5′MECOMx2)[188/200]
94	F	75	t-AML	A, PR	2	45,XX,t(3; 12)(q26.2; p13),-7,idic(22)(p11.2)[16]/46,XX[4]	nucish(MECOMx2)(3′MECOM sep 5′MECOMx1)[107/200]
95	M	59	CML, BP	D	12	49,XY,t(3; 12)(q26.2; p13),+8,+8,+i(8)(q10),i(8)(q10)x2,t(9; 22)(q34; q11.2)[19]/46,XY	ish t(3; 12)(q26; p13)(5′ETV6+; 3′ETV6+,5′ETV6-)[3].nucish(MECOMx2)(3′MECOM sep 5′MECOMx1)[104/200]
96	M	28	AML	A, PR	0	45,XY,t(3; 12)(q26.2; p13),del(5)(q22q33),-7[20]	ish t(3; 12)(q26.2; p13)(3′MECOM+; 5′MECOM+)[1].nucish(MECOMx2)(3′MECOM sep 5′MECOMx1)[82/200]
97	M	32	CML, BP	D	6	46,XY,t(3; 17)(q26.2; q22),t(9; 22)(q34; q11.2)[20]	ish t(3; 17)(3′MECOM+; 5′MECOM+)[4].nucish(MECOMx2)(3′MECOM sep 5′MECOMx1)[132/200]
98	F	44	CML, BP	D	4	46,XX,t(3; 17)(q26.2; q22),t(9; 22)(q34; q11.2)[19]/47,idem,+der(22)t(9; 22)[1]	ish t(3; 17)(q26.2; q22)(MECOM+; 5′MECOM+)[4].nucish(3′MECOMx2,5′MECOMx3)(3′MECOM con 5′MECOMx2)[183/200]
99	M	77	t-MDS/AML, MM,	D	17	45,XY,der(1)t(1; 17)(p36.1; q25),t(3; 17)(q26.2; q23),-7,del(20)(q13.1q13.3)[26]	ish t(3; 17)(5′MECOM+; 3′MECOM+)[3].nucish(MECOMx2)(3′MECOM sep 5′MECOMx1)[172/200]
100	F	47	t-MDS	D	3	46,XX,add(3)(q27),t(3; 17)(q26.2; q23)[3]/46,XX[17]	ish add(3)(q27)(MECOM+),t(3; 17)(q26.2; q23)(5′MECOM+; 3′MECOM+)[1].nucish(MECOMx2)(3′MECOM sep 5′MECOMx1)[150/200]
101	M	22	AML	D	10	47,XY,+X?c,t(3; 21)(q26.2; q22),del(13)(q12q14)[20]	ish t(3; 21)(q26.1; q22)(3′MECOM+,5′MECOM+)[2].nucish((MECOMx2)(3′MECOM sep 5′MECOMx1)[188/200]nucish(MECOMx2)[200]nucish(MECOMx2)(3′MECOM sep 5′MECOMx1)[178/200]
102	M	75	MDS + MCL	D	7	46,XY,r(7)(p11.2q11.2),der(12)del(12)(p11.2p12)inv(12)(p13q21)[3]/46,idem,t(3; 21)(q26.2; q22)[2]/46,XY[2]	ish t(3; 21)(q26.2; q22)(5′MECOM+; 3′MECOM+)[1].nucish(MECOMx2)(3′MECOM sep 5′MECOMx1)[84/200]
103	F	59	MDS	D (SCT)	12	46,XX,t(3; 21)(q26.2; q22)[3]	ish t(3; 21)(3′MECOM+; 5′MECOM+).nucish(MECOMx2)(3′MECOM sep 5′MECOMx1)[104/200]
104	M	65	AML	D	9	46,XY,t(9; 17)(p22; q25),t(15; 15)(q21; q26)[7]/46,XY,idem,inv(3)(p25q12)[1]/48,XY,t(3; 21)(q26.2; q22),+13,+14[3]/46,XY[9]	ish t(3; 21)(q26.2; q22)(3′MECOM+; 5′MECOM+)[3].nucish(MECOMx2)(3′MECOM sep 5′MECOMx1)[17/200]
105	M	82	AML/MDS	D	8	47,XY,t(3; 21)(q26.2; q22),+8[18]/48,idem,+mar[2]	ish t(3; 21)(q26.2; q22)(3′MECOM+; 5′MECOM+)[5].nucish(MECOMx2)(3′MECOM sep 5′MECOMx1)[160/200]
106	M	67	MDS	D	5	46,XY,t(3; 21)(q26.2; q22),add(7)(q22)[13]/46,XY[7]	ish t(3; 21)(q26.2; q22)(3′MECOM+; 5′MECOM+)[3].nucish(MECOMx2)(3′MECOM sep 5′MECOMx1)[59/200]
107	F	80	AML	D	15	46,XX,t(3; 21)(q26.2; q22)[1]/46,idem,t(2; 7)(q36; p15)[14]/46,XX[5]	nucish(3′MECOMx2,5′MECOMx3)(3′MECOM con 5′MECONx2)[191/200]
108	M	46	AML	D	2	46,XY,t(3; 21)(q26.2; q22),der(7)t(1; 7)(q21; q22)[6]/46,idem,+der(13; 21)(q10; q10),der(13; 21)(q10; q10)[14]	ish t(3; 21)(q26.2; q22)(MECOM+; 5′MECOM+)[4].nucish(3′MECOMx2,5′MECOMx3)(3′MECOM con 5′MECOMx2)[181/200]
109	M	78	AML	D	10	46,XY,der(7; 18)(p10; q10),+11[2]/46,idem,t(3; 21)(q26.2; q22)[5]/45,X,-Y[5]/46,XY[8]	ish t(3; 21)(q26.2; q22)(MECOM+; 5′MECOM+)[4].nucish(3′MECOMx2,5′MECOMx3)(3′MECOM con 5′MECOMx2)[50/200]
110	M	75	AML	A, NR	6	46,XY,t(3; 21)(q26.2; q22)[6]45,idem,-7[4]/46,XY[10]	nucish(MECOMx2)(3′MECOM sep 5′MECOMx1)[163/200]
111	F	63	MDS	A, PR	5	46,XX,t(3; 21)(q26.2; q22)[8]/46,idem,del(11)(p11.2)[5]/46,XX[4]	ish t(3; 21)(q26.2; q22)(MECOM+; 5′MECOM+)[2].nucish(3′MECOMx2,5′MECOMx3)(3′MECOM con 5′MECOMx2)[173/200]
112	F	69	MDS, AML	D	0	46,XX,t(3; 21)(q26.2; q22),del(5)(q15q33),inv(9)(p12q13)[20]	nucish(MECOMx2)(3′MECOM sep 5′MECOMx1)[175/200]
113	F	53	t-MDS/AML	A, PR	3	45,XX,t(3; 21)(q26.2; q22),-7[20]	nucish(3′MECOMx2,5′MECOMx3)(3′MECOM con 5′MECOMx2)[40/200]
114	M	71	AML/MDS	A, NR	2	46,XY,t(3; 21)(q26.2; q22)[15]/46,XY[5]	ish t(3; 21)(q26.2; q22)(MECOM+; 5′MECOM+)[3].nucish(3′MECOMx2,5′MECOMx3)(3′MECOM con 5′MECOMx2)[173/200].nucish(RUNX1T1x2,RUNX1x3)[165/200]
115	M	39	t-MDS	A, PR	2	46,XY,t(3; 21)(q26.2; q22)[5]/46,idem,add(10)(p12)[1]/46,idem,inv(11)(p15q23)[1]/46,XY[13]	nucish(3′MECOMx2,5′MECOMx3)(3′MECOM con 5′MECOMx2)[176/200]
116	M	63	AML	D	7	46,XY,add(2)(p21),-3,-3,del(5)(q13q33),+8,add(17)(p11.2),add(22)(q13),+der(?)ins(?; 3)(?; q26.2q26.2)[3]/47,idem,+16[6]/45∼57,XY,add(2)(p21),-3,-3,del(5)(q13q33),+8,add(11)(p15),+16,add(17)(p11.2),add(22)(q13),+1∼5mar[cp11]	ish der(17)t(3; 17)(q22; p12)(MECOM+),der(?)ins(?; 3)(?; q26.2q26.2)(MECOM dim)[2]/der(17)t(3; 17)(q22; p12)(MECOM+),der(?)ins(?; 3)(?; q26.2q26.2)(3′MECOM+)[1].nuc ish(MECOMx1,MECOM dimx1)[463/500]/(3′MECOMx2,5′MECOMx1)(3′MECOM con 5′MECOMx1)[19/500]
117	F	69	AML	D	10	45,XX,der(1)ins(1; 3)(p22; q24q26.2)del(1)(q32),der(3)ins(1; 3)(p22; q24q26.2),del(5)(q22q33),-7,add(10)(p12)[19]/46,XX[1]	ish der(1)ins(1; 3)(p22; q24q26.2)del(1)(q32)(3′MECOM+),der(3)ins(1; 3)(3′MECOM-,5′MECOM+)[2].nuc ish(MECOMx2)(3′MECOM sep 5′MECOMx1)[194/200]
118	M	18	AML	D	2	38∼44,X,-Y,i(1)(q10),add(2)(p23),der(3)dup(3)(q23q27)add(3)(q29),-5,del(5)(q22q35),der(7)t(1; 7)(q21; p15),-9,add(10)(q22),add(15)(p11.2),der(16)t(?; 16)(?; p12)ins(?; 3)(?; q26.2q26.2),add(17)(p11.2),-18,-19,-21,+1∼3mar[cp20]	ish der(3)dup(3)(q23q27)(MECOM++)add(3)(q29), der(16)t(?; 16)(?; p12)ins(?; 3)(?; q26.2q26.2)(5′MECOM+)[2].nucish(3′MECOMx3,5′MECOMx4)(3′MECOM con 5′MECOMx3)[189/200]
119	M	53	AML	D	6	51,XY,del(3)(q26.2),+8,der(8)ins(8; 3)(q24.1; q26.2q26.2),t(9; 22)(q34; q11.2),add(11)(q23),+19,+20,+21,+der(22)t(9; 22)[20]	ish del(3)(q26.2)(3′MECOM+),der(8)ins(8; 3)(q24.1; q26.2q26.2)(5′MECOM+)[4].nuc ish(MECOMx2)(3′MECOM sep 5′MECOMx1)[195/200]
120	F	73	MDS	A, PR	7	46,XX,ins(12; 3)(p13; q21q26.2)[11]/46,XX[9]	ish ins(12; 3)(p13; q21q26.2)(MECOM+; 5′MECOM+)[2].nucish(3′MECOMx2,5′MECOMx3)(3′MECOM con 5′MECOMx2)[64/200].nucish(5′ETV6x2,3′ETV6x1)(5′ETV6 con 3′ETV6x1)[60/200]
121	F	39	AML	D	12	45,XX,ins(3; 3)(q21; q21q26.2),-7[14]//46,XY,inv(9)(p12q13)[6]	ish ins(3; 3)(q21; q21q26.2)(3′MECOM+,MECOM+; 5′MECOM+)[2].nucish(MECOMx2)(3′MECOM sep 5′MECOMx1)[79/200]
122	M	70	AML	D	1	41∼44,XY,der(3)del(3)(q25q26.2)add(3)(q27),der(3)del(3)(q25q26.2)hsr(3)(q26.2),-4,-5,-6,add(7)(p15),-12,-17,-21,add(22)(q11.2),+der(?)ins(?; 3)(?; q26.2q29)dup(3)(q26.2q29),+1∼3mar[cp20]	ish der(3)del(3)(q25q26.2)add(3)(q27)(3′MECOM-,5′MECOM+),der(?)ins(?; 3)(?; q26.2q29)(5′MECOM+)dup(3)(q26.2q29)(5′MECOM+)[1]/der(3)del(3)(q25q26.2)hsr(3)(q26.2)(3′MECOM-,5′MECOM amp)[2].nucish(MECOMx1,5′MECOM amp)[48/200]/(3′MECOMx1,5′MECOMx3∼6)(3′MECOM con 5′MECOMx1)[90/200]
123	M	50	AML	D	7	44,XY,add(4)(p16),del(5)(q13q33),add(8)(p21),add(8)(q24),add(17)(p12),-18,-19,-20,+mar[12]/44,XY,add(4)(p16),del(5)(q13q33),add(8)(q24),add(17)(p12),-18,-19,-20,+22[4]/42∼45,XY,add(4)(p16),del(5)(q13q33),add(8)(p21),add(8)(q24),add(17)(p12),-18,-19,-20,+1∼3mar[cp4].	nucish(3′MECOMx2,5′MECOMx3)(3′MECOM con 5′MECOMx2)[40/200]
124	M	78	AML	A, PR	0	45,XY,-7[19]/46,XY[1] (**cryptic**)	nucish(MECOMx2)(3′MECOM sep 5′MECOMx1)[42/200]
125	F	69	AML	D	1	46,XX,add(3)(q21),inv(5)(p15q31)[20]	ish add(3)(q26)(3′MECOM+; 5′MECOM-)[2].nucish(3′MECOMx2,5′MECOMx1)(3′MECOM con 5′MECOMx1)[150/200]
126	M	74	CMML	D	9	46,XY,del(3)(q21q25)[5]/47,XY,+12[1]/46,XY[14]	ish del(3)(q21q25)(MECOM+)[2].nucish(3′MECOMx1,5′MECOMx2)(3′MECOM con 5′MECOMx1)[5/200]
127	M	54	AML	D	7	43-44,XY,-3,-4,add(5)(q11.2),-20,add(21)(q22),-22,+r,+1-2mar[cp20]	ish r(3′MECOM+,3′MECOM+,5′MECOM+,5′MECOM+)[3].nucish(MECOMx3)(3′MECOM sep 5′MECOMx2)[183/200]
128	F	18	t-MDS	D	0	n/a	nucish(MECOMx2)(3′MECOM sep 5′MECOMx1)[47/200]
129	M	60	**MM;** MDS	A, PR(SCT)	27	41,X,-Y,+1,der(1)t(1; 14)(p13; q12)t(14; 16)(q32; q23),del(1)(p34),del(1)(q22),t(3; ?)(q26.2; ?),del(6)(q14),der(7)t(7; 8)(p21; q12),add (7)(q36),del(8)(p22),-9,dic(9; ?)(q34; ?)t(?; 11)(?; q12),psu dic(10; 1)(q26; q12),-11,del(11)(q12),-13,-14,-15,-15,der(16)t(14; 16),add(16)(q12),-17,+19,add(19)(p13),add(21)(p13)x2,-22,+1∼4mar[cp14]/46,XY[6]	nucish(3′ MECOMx2,5′ MECOMx3)(3′ MECOM con 5′ MECOMx2)(MECOM amp)[100/200]

MM: multiple myelom; cryptic: can't be detected by chromosomal analysis

## 2 Experimental design, materials and methods

This is a retrospective study. A search of the cytogenetics database for *MECOM* FISH positive cases from May 1, 2009 to August 15, 2018 at The University of Texas MD Anderson Cancer Center (MDACC) has been performed, and a total of 129 cases with at least one time *MECOM* FISH positive result has been found. Other cases with 3q26 abnormality by conventional cytogenetics but negative for and/or not been confirmed for *MECOM* rearrangement are excluded from this study. Clinicopathologic data, including *MECOM* FISH test results, were collected by electronic medical chart review.

Conventional G-banded chromosomal analysis (karyotyping) has been routinely performed on unstimulated 24-h and 48-h BM aspirate cultures using standard techniques as we have reported previously [6], [7], [8]. Interphase-, metaphase-, map-back FISH and WCP have been performed by following existing laboratory protocols as reported previously [6], [7], [8], [9]. The following probe/probe set have been employed for this study. The *MECOM (EVI1)* dual color, breakapart (BAP) DNA probe (#KI-10204) from Leica Biosystems/Kreatech (Buffalo Grove, IL; theVysis LSI *BCR/ABL* ES Dual Color Fusion probe, Vysis*MYC* BAP probe; the Vysis ETV6 BAP Probe and the Vysis*RUNX1T1/RUNX1* Fusion probe (Abbott Molecular, Des Plaines, IL).All these probe/probe set used for our clinical diagnosis have been validated in our laboratory in accord with the American College of Medical Genetics and Genomics (ACMGG) guidelines.
