# Supplementary material for: Data on MECOM rearrangement-driven chromosomal aberrations in myeloid malignancies
Source: Data Brief. 2019 May 23;24:104025. doi: 10.1016/j.dib.2019.104025 (PMC6545385; doi:10.1016/j.dib.2019.104025)
Supplement: Multimedia component 1 [file mmc1.docx]

**Statement of conflict of interest**

All authors declare that there is no conflict of interest.
